# Transport networks and inequities in vaccination: remoteness shapes measles vaccine coverage and prospects for elimination across Africa

**DOI:** 10.1017/S0950268814001988

**Published:** 2014-08-14

**Authors:** C. J. E. METCALF, A. TATEM, O. N. BJORNSTAD, J. LESSLER, K. O'REILLY, S. TAKAHASHI, F. CUTTS, B.T. GRENFELL

**Affiliations:** 1 Department of Zoology, Oxford University, Oxford, UK; 2 Fogarty International Center, National Institute of Health, Bethesda, MD, USA; 3 Department of Ecology and Evolutionary Biology, Eno Hall, Princeton University, Princeton, NJ, USA; 4 Department of Geography and Environment University of Southampton, Southampton, UK; 5 Flowminder Foundation, Stockholm, Sweden; 6 Centre for Infectious Disease Dynamics, The Pennsylvania State University, University Park, PA, USA; 7 Department of Epidemiology, John Hopkins Bloomberg School of Public Health, Baltimore, MD, USA; 8 Medical Research Council Centre for Outbreak Analysis and Modelling, Department of Infectious Disease Epidemiology, School of Public Health, Imperial College London, London, UK; 9 London School of Hygiene and Tropical Medicine, London, UK

**Keywords:** Epidemiology, modelling, measles (rubeola), rubella, vaccine policy development

## Abstract

Measles vaccination is estimated to have averted 13·8 million deaths between 2000 and 2012. Persisting heterogeneity in coverage is a major contributor to continued measles mortality, and a barrier to measles elimination and introduction of rubella-containing vaccine. Our objective is to identify determinants of inequities in coverage, and how vaccine delivery must change to achieve elimination goals, which is a focus of the WHO Decade of Vaccines. We combined estimates of travel time to the nearest urban centre (⩾50 000 people) with vaccination data from Demographic Health Surveys to assess how remoteness affects coverage in 26 African countries. Building on a statistical mapping of coverage against age and geographical isolation, we quantified how modifying the rate and age range of vaccine delivery affects national coverage. Our scenario analysis considers increasing the rate of delivery of routine vaccination, increasing the target age range of routine vaccination, and enhanced delivery to remote areas. Geographical isolation plays a key role in defining vaccine inequity, with greater inequity in countries with lower measles vaccine coverage. Eliminating geographical inequities alone will not achieve thresholds for herd immunity, indicating that changes in delivery rate or age range of routine vaccination will be required. Measles vaccine coverage remains far below targets for herd immunity in many countries on the African continent and is likely to be inadequate for achieving rubella elimination. The impact of strategies such as increasing the upper age range eligible for routine vaccination should be considered.

## INTRODUCTION

Vaccination has proved one of the most successful public health interventions, resulting in substantial mortality and morbidity reductions worldwide [1, 2]. Between 2000 and 2012, it was estimated that vaccination averted 13·8 million deaths [3], and future impacts are anticipated to be large [4]. However, despite major successes in ramping up vaccination [5], significant outbreaks occurred across the African continent between 2009 and 2011 [6]; and many individuals remain at risk. Both low coverage of routine vaccination programmes, and suboptimal implementation of the catch-up or follow-up campaigns designed to reach susceptible children (Supplementary Immunization Activities, or SIAs) who had accumulated over the previous years of low routine coverage [7] contribute to this. Nevertheless, all six World Health Organization (WHO) regions currently have measles elimination goals [8]. The impact of inequities in vaccination rates, and the resulting heterogeneity in the population landscape of immunity on the measles endgame, is consequently a key public health question. A related issue is how these heterogeneities affect the prospect of introduction of rubella-containing vaccine, which can have negative impacts if vaccine coverage is inadequate. Here, we show that geographical isolation plays a key role in shaping vaccination inequity across a range of countries in Africa and explore how modalities for enhancing vaccination coverage will impact geographical inequity.

The considerable variability in the opportunity that children have for vaccination is well-recognized in the literature [9]. Various correlates of low coverage have been suggested, linked to health service availability and performance [10], socio-demographic characteristics of families and communities [11, 12], and their perceptions and attitudes to vaccination [13–15]. A range of work suggests that geographical location may be important [16–18], usually linked to access to care [10], with particular emphasis on urban/rural differences [14, 19, 20]. For example, coverage in the northern states of Nigeria is about half of that in the southern states, and lower in rural than urban areas [21]. However, there are also exceptions, e.g. in Kenya, travel time to vaccine clinics did not have any discernible impact on coverage [22]. Such geographical variation is of particular public health relevance for measles, as geographical clustering of unvaccinated individuals may be crucial in allowing persistent circulation of the virus [23]. Rubella vaccine, until recently rarely used in low-income countries [24], is being introduced with increasing frequency since funding for its introduction became available via the Global Alliance for Vaccines and Immunization (GAVI) [25]. Rubella vaccination is easily combined with the measles vaccine, and existing measles programmes are consequently the likely delivery mechanism if the vaccine is introduced [26]. Consequently, measles coverage levels are informative for the advisability of rubella introduction. Inequities in coverage are also particularly significant for rubella control [27]. Areas of low coverage that are of insufficient size to maintain circulation of rubella (i.e. are below the critical community size [28]) may be particularly vulnerable to accumulation of late age susceptible individuals [27] and thus increase the main burden of rubella linked to infection of women during early pregnancy [29].

Here we systematically analyse the impact of geographical isolation on coverage of the first dose of measles-containing vaccines (MCV1) in Africa. We show a link between coverage and average per person travel time (across available modes of transport) to population centres of ⩾50 000 people. We use our analysis to assess the penetration of routine vaccination into rural communities, and to investigate modified scenarios of vaccine delivery, including changing the rate of delivery, changing the target age range of delivery, or reducing travel-linked inequities to identify which most effectively move populations towards the 95% vaccination coverage estimated as a threshold for measles elimination [30]. We also quantify the magnitude of change necessary in remote populations to achieve 80% coverage, considered to be a key threshold for introduction of rubella vaccination [26] (although note that this may be too low in some circumstances [31, 32]).

## METHODS

### Vaccination coverage data

Measles vaccination status, age, and approximate geographical longitudes and latitudes of residence for children aged 9–59 months were obtained from Demographic Health Surveys (DHS) on the African continent occurring during or after 2000 [33]. These surveys select a large random sample of households to provide nationally, and sometimes regionally, representative estimates of key demographic and health indicators, including vaccination coverage [33]. In reporting the geographical coordinates (standard latitude and longitude values [34]), points are displaced to protect the confidentiality of the survey respondents.

In the course of surveys, mothers were asked whether the child had a vaccination card; and additionally whether ‘the child had a measles injection or an MMR injection – i.e. a shot in the arm at the age of ⩾9 months – to prevent him/her from getting measles?’ [33]. We used the latter to develop an indicator variable defining whether children had ever been vaccinated (taking the value 1 if mothers reported positively, 0 if mothers reported negatively, and NA if mothers did not report). Although the number of measles vaccine doses received by children is also of considerable public health relevance (WHO recommends that each child should receive two doses, because of interference from maternal immunity), with the data available, we could not quantify this over the broad scales of interest, so we focused on presence/absence of vaccination.

Supplementary Table S1 provides numbers of children for which vaccination and age information was available per country and country-specific details of SIAs based on WHO data. Given the lack of exact dates of delivery of vaccines during SIAs and the difficulty in determining the age of many children in countries without birth registration, the age ranges assumed to be eligible during each survey are approximate. The coverage reported by mothers does not distinguish between whether vaccination was provided as part of routine or SIA campaigns, so we were unable to disentangle the roles of these different modes of immunization.

### Remoteness data

Spatial datasets on Africa-wide road networks (GRoads: www.ciesin.columbia.edu/confluence/display/roads/; VectorMap0: www.mapability.com; OpenStreetMap: www.openstreetmap.org; plus national transportation network GIS datasets from Kenya, Namibia, Tanzania, Swaziland, Rwanda, Niger, Zambia, Angola, Somalia and Djibouti), land cover [35], settlement locations [36] (www.worldpop.org.uk), inland water bodies [37] and topography [38] were obtained and assembled within a geographical information system (GIS). The datasets were the most detailed and complete available, and all were constructed within the last 10 years to represent as closely as possible conditions during the period within which the DHS surveys were conducted. These datasets formed the basis for constructing a ‘friction’ surface used to calculate travel times to the nearest settlement of ⩾50 000 people in 2010, following the methodology outlined by Nelson [39].

The gridded travel time dataset was used to map out areas at travel times of 1-h intervals (0–1 h, >1–2 h, etc., up to >10 h) from settlements of ⩾50 000 population, which we refer to as ‘urban’. These mapped classes were then overlaid onto a 2010 gridded population dataset [36] and the population sizes residing in each class for each country were calculated. We use travel time from large urban centres rather than, say, distance to the nearest health facility because travel time data is broadly available and comparable across countries whereas health facility data is more variable.

### Model fitting

We first explored various parametric hazard regression models for coverage as a function of age and distance. However, no such models adequately captured the varying patterns seen across the continent. We therefore used non-parametric binomial regression to estimate the probability of vaccination as a function of age and remoteness using local polynomial regression with a logit link and a binomial error [40]. Local polynomials are akin to regression splines but are better able to accommodate unequally spaced data (e.g. [41]). For each country/year, the model was fit to data from all children for whom vaccination data, age, and location were available. We combined this with data on population numbers found across travel-time classes to estimate country-level coverage and compared this with previous DHS scaled estimates (obtained via the DHS website, http://www.statcompiler.com) to validate the models.

### Kinetics of vaccination over age and geography

We explored the impact of a range of different scenarios on country-level measles vaccine coverage. First, we quantified what increase in coverage may be obtained by improving rates of routine vaccination, assuming that within each travel time category (taken in hourly increments) rates of vaccination attained over 1 week between 9 and 12 months increased by half the baseline rate [see Supplementary Fig. S1: for example, if baseline coverage increased from 50% to 51% with one additional week of age, then coverage was assumed to increase by 0·15 = 1·5 × (0·51–0·5) over the same week of age]. Second, we quantified what increase in coverage may be obtained by increasing the upper age range considered for routine vaccination. To do this, we assumed that the maximum rate of vaccine delivery attained between 9 and 10 months represented coverage achieved under routine vaccination, and explored the impact if this rate of delivery was applied for ages up to 15 months (see Supplementary Fig. S1 for a representation of this modification; broadly similar results are obtained if the value is applied up to 18 months). Third, we quantified what increase in coverage may be achieved by removing urban/rural inequities, i.e. assuming that all rural areas would get the same access to vaccination as urban locations in that country. For all these comparisons, we focused on coverage attained at 24 months (since increasing the age range will show no impact at 12 months).

Throughout, institutional ethics approval was not sought because this is a retrospective study and the databases are anonymized and free of personally identifiable information.

## RESULTS

**Fig. 1.**
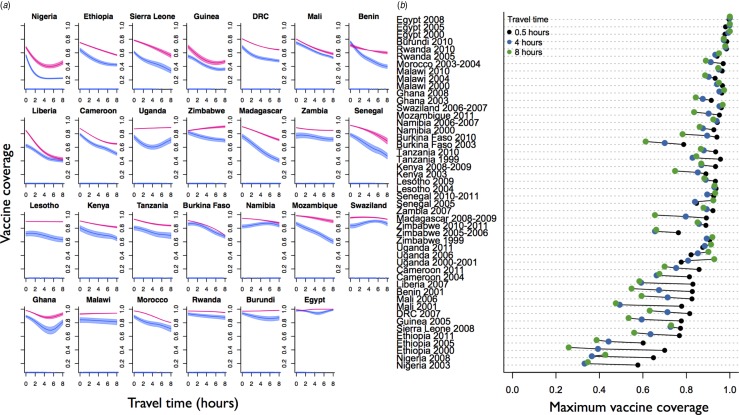
The effect of travel time on vaccination coverage. (*a*) The proportion of the population vaccinated (y axis) achieved between 12 and 13 months of age (blue areas) and 58 and 60 months of age (pink polygons) as a function of travel time in hours to the nearest city of ⩾50 000 people (x axis) for the most recent Demographic Health Survey (DHS) available from each country. (*b*) The maximum proportion of the population vaccinated by age 60 months at 0·5, 4, and 8 h travel time (legend, colours) for the full range of available DHS data available for each country, ordered by coverage achieved in the most recent DHS survey. For the approximate age range eligible for Supplementary Immunization Activities and where they occurred, see Supplementary Table S1.

Figure 1*a* shows the distance effect on measles vaccine coverage achieved by 13 months and 60 months from the most recent survey for all countries for which data was available (shown respectively in pink and blue, see Supplementary Fig. S2 for detailed plots of all ages). These ages were chosen since most children are likely to have received their routine dose by 13 months; SIAs may increase the coverage observed at 60 months, which also reflects the upper age available with the data. The remoteness effect for the full range of available data is shown in Figure 1*b* (see Supplementary Fig. S3 for how travel times map onto the various countries). Both plots indicate a clear signal of urban/rural inequity in most countries, and Figure 1*b* shows that the inequity is amplified in countries with lower overall coverage. Estimates of country-level coverage across the dataset concur with estimates from the DHS (Supplementary Fig. S4), indicating that our mapping from coverage across age and travel time classes combined with population distribution across travel time classes provides a reasonable reflection of the overall coverage for the different countries.

**Fig. 2.**
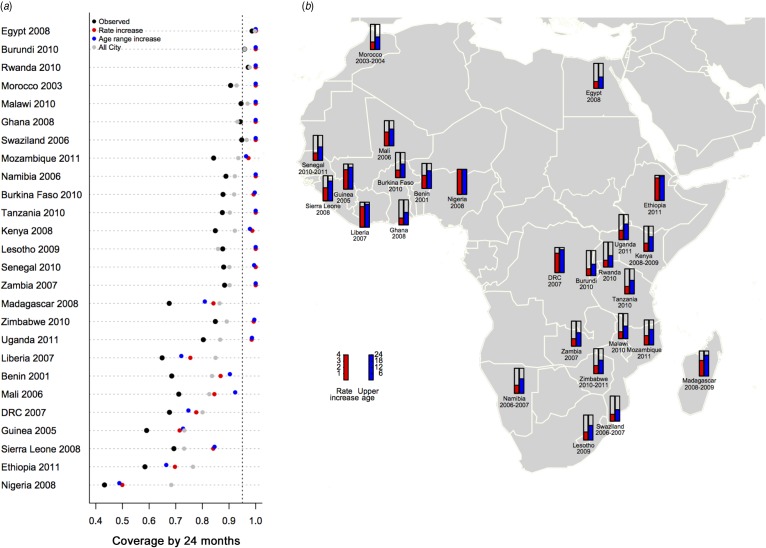
Impact of modifying kinetics of measles vaccination coverage by country. (*a*) Vaccination coverage by country achieved at 24 months (black points), obtained by scaling estimated coverage by the size of populations living at different travel times (assuming an even distribution of population across ages up to age 5 years); the coverage level by 24 months that would be obtained if the rate of vaccination between 9 and 12 months could be increased by 50% (red points); or if the age range whereby the maximum rate of coverage was obtained was extended up to 15 months (blue points); or everyone obtained the coverage estimated in the larger urban centres (grey points). Results are ordered by maximum coverage obtained over age (see Fig. 1). (*b*) The factor by which the rate of vaccination between 9 and 12 months would need to be multiplied to achieve 95% coverage by 24 months (red) and the degree to which the upper age of vaccination must be increased to achieve 95% coverage by 24 months (blue) shown to reflect geographical clustering.

The analysis of modification of the kinetics of vaccination (Fig. 2*a*) indicates that all three scenarios would increase coverage but their relative effectiveness is country-specific. In those countries with the most to gain in vaccination coverage (baseline <0·70), the greatest benefits would come from removing ‘rural penalty’ (grey circles, Fig. 2*a*; coverage raised by between 3 and 25 percentage points and on average increased by 16 percentage points). However, with the exception of a few of the countries that currently report coverage >90%, removing rural penalty alone would be insufficient to raise coverage over the 95% elimination threshold. For this additional adjustments such as increased rate of vaccination during routine, or an increased target age range (which by increasing the window of time for vaccination can substantially increase coverage by 24 months) would be required (see Supplementary Fig. S5A for the ratio of extra doses deployed that this corresponds to). Figure 2*b* lays out the various country-specific scenarios required to reach 95% coverage; the red bars indicate the factor by which the rate of routine vaccination must be multiplied; and the blue bars indicate the increased age limit required given current uptake rates. The corresponding increase in the number of doses administered for rate- or age-range increase scenarios (Supplementary Fig. S5B) tends to be rather similar, as one would expect. Note that in these illustrations, the various kinetic changes are independent and considered separately.

**Fig. 3.**
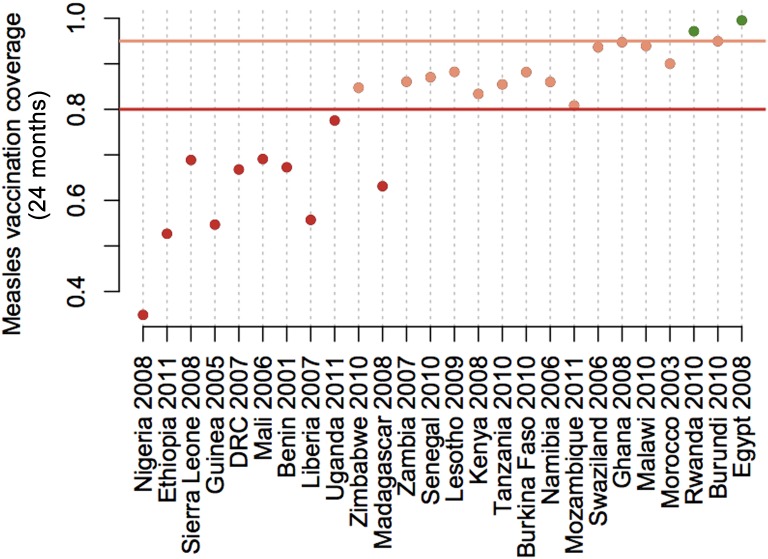
Shortfall in measles vaccination coverage in remote communities. The y axis indicates the level of measles vaccination coverage attained at 24 months for children living at travel times reflecting the 0·75 quartile of the travel time (i.e. the most remote children) for countries shown on the x axis. Distance below the horizontal lines indicate the necessary increase in measles vaccination coverage in the least served communities required to achieve values >80% in every community (dark red line, suggested minimum for safe introduction of rubella-containing vaccine) or >95% in every community (lighter red line, suggested level required to achieve measles elimination).

Countries with the lowest coverage also have the largest shortfall to achieve either measles elimination targets (95%), or minimal levels of coverage for rolling out rubella containing vaccine currently recommended by WHO (80%) in the most remote communities. Coverage achieved in the most remote communities relative to these two thresholds is shown in Figure 3.

## DISCUSSION

Reducing inequity in vaccine coverage is a key strategic objective of the Decade of Vaccines [42]. The 2013 Action Plan [42] called for strategies to go beyond the 2002 concept of ‘Reaching Every District’ to encompass ‘Reaching Every Community’. This Action Plan emphasized that implementing such strategies requires that the underserved be identified; and that progress in accessing this group be monitored [42]. Our analysis provides a simple predictive variable of vaccine-related inequity: vaccination coverage is lower in more remote locations as measured by average per person travel time. Furthermore, due to its simplicity, this metric can easily be re-evaluated to assess progress towards equity goals.

Countries for which the effect of remoteness on measles vaccine coverage is relatively small tend to be those with overall high measles vaccine coverage (e.g. Egypt or Ghana). In many countries with repeat surveys, an increase in coverage has been accompanied with a marked reduction in inequity. For example, in Ghana between 2003 and 2008, Kenya between 2003 and 2008, and Namibia between 2000 and 2006 the ‘rural penalty’ is considerably reduced (Fig. 1*b*), suggesting that strategies to reach more communities have been successful. However, there are exceptions to this pattern (e.g. Burkina Faso 2003–2010). The mechanisms underlying this inequity, and how it changes in the face of improved global coverage are likely to be broadly linked to infrastructure, although geographic variation in socioeconomic status, ethnic group and other family and community characteristics may also play a role [11, 12]. How these factors can be mitigated via design of delivery of routine and supplementary immunization activities will be an additional important issue in reaching target vaccine coverage. SIAs in particular are intended specifically to redress this type of inequity by reaching children in remote and underserved communities [8]. The data available here were not sufficient to disentangle the role of SIAs *vs.* routine vaccination since the data report on whether or not children were vaccinated, but not the means by which the vaccine had been distributed. A key research question is to establish the degree to which SIAs reduce inequities, particularly across spatial scales.

Our analysis of possible changes to the kinetics of immunization, including increasing the rate of vaccination, and increasing the upper age to which routine vaccination is applied appear to have potentially large effects on measles coverage. Most interestingly, increasing the upper age of eligibility for routine-like vaccination is predicted to have substantial effects, a result of considerable interest as this change in control does not require any changes in infrastructure. However, some countries remain below the threshold of herd immunity for measles even with these changes (Liberia, Benin, Mali, DRC, Guinea, Sierra Leone, Ethiopia, Nigeria). It is key to acknowledge that other heterogeneities will affect these results – in our scenario analyses, we assume that individuals of the same age and at the same travel time have an equal opportunity of vaccination. In reality, there may be parts of local populations that has greatly reduced opportunities for vaccination [43]. Increasing rates of vaccination, or the upper age of routine vaccination will be less likely to affect such strata.

Finally, as we approach measles elimination, achieving high coverage not just at the scale of the country, but also in pockets where infection is likely to persist is of increasing importance. Various lines of evidence suggest that remote communities may have lower *R*_0_, as transmission rate is thought to increase with density of contacts [44, 45]. For measles, the lower the *R*_0_, the lower the critical threshold of vaccination required for elimination. However, this remains relatively poorly understood – for example, seasonal fluctuations and aggregation might also be higher in such areas, potentially driving a very high seasonal peak of transmission [45]. Whatever the case, the impact of persisting pockets may considerably complicate and extend the duration of the endgame [46]. For rubella, lower levels of *R*_0_ also may lead to higher probability of local extinction of the virus in remote communities, and thus greater potential for build-up of late-age susceptible individuals [47]. The pattern of coverage shortfalls in remote communities (Fig. 3) reflects that of the pattern of coverage in general, once again emphasizing that reaching these remote communities is key.

Inequities in vaccine coverage are often quantified at broad spatial scales (e.g. provinces, countries) or in terms of gender, or socioeconomic indicators. Finer scale analyses of healthcare accessibility require considerably more data, and thus are often narrower in scope. Our analysis combines broad scale data with a relatively simple measure of accessibility to address this question. Evidence for remote under-vaccinated populations is both of considerable importance given the dynamical consequences of unvaccinated communities, but also as another simple indicator of where underserved populations are to be found, with the potential to guide policy to address their needs.

## SUPPLEMENTARY MATERIAL

For supplementary material accompanying this paper visit http://dx.doi.org/10.1017/S0950268814001988.
