# Supplementary material for: Transport networks and inequities in vaccination: remoteness shapes measles vaccine coverage and prospects for elimination across Africa
Source: Epidemiol Infect. 2014 Aug 14;143(7):1457–66. doi: 10.1017/S0950268814001988 (PMC4411642; doi:10.1017/S0950268814001988)
Supplement: Supplementary file 1 [file S0950268814001988sup.zip › S0950268814001988sup006.docx]

**Figure S1:** Vaccination scenarios explored**.** From a baseline pattern of probability of vaccination over age (black line corresponding to a particular travel time context) we explored the impact of increasing the rate of vaccination between 9 and 12 months (the slope of the red line is 1.5 that of the black line between 9 and 12 months) and the impact of increasing the upper age at which maximal vaccination rates are achieved to 15 months (the slope of the blue line retains the values achieved between 9 and 10 months up to 15 months).

**Figure S2**: Measles vaccination coverage (z axis) as a function of travel time in hours to the nearest city of 50,000 or more people (x axis) and age in months (y axis) fitted using local polynomials; for all country/year combinations available in the DHS.

**Figure S3:** Map showing travel times depicted in Figure 1b across the African continent; colours as in Figure 1b.

**Figure S4**: Comparison of DHS vaccination coverage estimates (x axis) with coverage obtained at the country level in 12 month olds (combining estimates of coverage at a range of travel times with population distribution across travel times). Colours of the dots indicate year of survey.

**Figure S5**: The ratio of doses deployed for the various scenario analyses relative to that estimated for the DHS A) as in Figure 2A with increased rates of vaccination between 9 and 12 by 1.5 (red points); and increased age range up to 15 months (blue points), and for the situation where the rural penalty is eliminated (grey points); and B) as in Figure 2B indicating the ratio of doses deployed for the rates (red) and age ranges (blue) required to achieve 95% coverage; elimination of the rural penalty also shown for comparison (grey).

**Table S1:** Details of the DHS data used, including number of children for which information on age, location, and vaccination status; and if an SIA occurred for which children present in the survey would have been eligible, the year, age range, and the eligible ages in months within the DHS data.

| **country** | **year of survey** | **N** | **SIA year** | **age targets** | | **eligible ages** | |
| --- | --- | --- | --- | --- | --- | --- | --- |
| Benin | 2001 | 247 | NA | NA | NA | NA | NA |
| Burkina Faso | 2003 | 400 | 1999 | 9 | 59 | 57 | 107 |
| Burkina Faso | 2003 | 400 | 2001 | 9 | 168 | 33 | 192 |
| Burkina Faso | 2010 | 573 | 2007 | 9 | 59 | 45 | 95 |
| Burkina Faso | 2010 | 573 | 2009 | 6 | 168 | 18 | 180 |
| Burundi | 2010 | 376 | 2006 | 9 | 59 | 57 | 107 |
| Burundi | 2010 | 376 | 2009 | 6 | 59 | 18 | 71 |
| DRC | 2007 | 300 | 2004 | 6 | 180 | 42 | 216 |
| DRC | 2007 | 300 | 2005 | 6 | 180 | 30 | 204 |
| DRC | 2007 | 300 | 2006 | 6 | 180 | 18 | 192 |
| DRC | 2007 | 300 | 2006 | 6 | 59 | 18 | 71 |
| Egypt | 2000 | 989 | NA | NA | NA | NA | NA |
| Egypt | 2005 | 1348 | 2002 | NA | NA | NA | NA |
| Egypt | 2008 | 1241 | NA | NA | NA | NA | NA |
| Ethiopia | 2000 | 539 | NA | NA | NA | NA | NA |
| Ethiopia | 2005 | 535 | NA | NA | NA | NA | NA |
| Ethiopia | 2011 | 596 | 2008 | 6 | 59 | 42 | 95 |
| Ghana | 2003 | 410 | 2002 | 9 | 168 | 21 | 180 |
| Ghana | 2008 | 408 | 2006 | 9 | 59 | 33 | 83 |
| Guinea | 2005 | 295 | 2003 | 9 | 168 | 33 | 192 |
| Kenya | 2003 | 399 | 1999 | 9 | 59 | 57 | 107 |
| Kenya | 2003 | 399 | 2002 | 9 | 168 | 21 | 180 |
| Kenya | 2008-2009 | 398 | 2006 | 9 | 59 | 33 | 83 |
| Kenya | 2008-2009 | 398 | 2006 | 9 | 59 | 33 | 83 |
| Lesotho | 2004 | 403 | 2000 | 60 | 168 | 108 | 216 |
| Lesotho | 2004 | 403 | 2003 | 9 | 59 | 21 | 71 |
| Lesotho | 2009 | 399 | 2007 | 9 | 59 | 33 | 83 |
| Liberia | 2007 | 298 | 2004 | 6 | 168 | 42 | 204 |
| Madagascar | 2008-2009 | 594 | 2004 | 9 | 168 | 57 | 216 |
| Madagascar | 2008-2009 | 594 | 2007 | 9 | 59 | 21 | 71 |
| Malawi | 2000 | 559 | 1998 | 108 | 168 | 132 | 192 |
| Malawi | 2004 | 521 | 2002 | 9 | 59 | 33 | 83 |
| Malawi | 2010 | 849 | 2008 | 9 | 59 | 33 | 83 |
| Mali | 2001 | 402 | NA | NA | NA | NA | NA |
| Mali | 2006 | 407 | 2004 | 9 | 59 | 33 | 83 |
| Morocco | 2003-2004 | 480 | NA | NA | NA | NA | NA |
| Mozambique | 2011 | 610 | 2008 | 9 | 59 | 45 | 95 |
| Namibia | 2000 | 258 | 1997 | 9 | 168 | 45 | 204 |
| Namibia | 2000 | 258 | 1998 | 9 | 59 | 33 | 83 |
| Namibia | 2006-2007 | 495 | 2003 | 9 | 59 | 45 | 95 |
| Nigeria | 2003 | 361 | NA | NA | NA | NA | NA |
| Nigeria | 2008 | 885 | 2005 | 9 | 180 | 45 | 216 |
| Nigeria | 2008 | 885 | 2006 | 9 | 180 | 33 | 204 |
| Rwanda | 2005 | 462 | 2003 | 9 | 168 | 33 | 192 |
| Rwanda | 2010 | 492 | 2006 | 9 | 59 | 57 | 107 |
| Rwanda | 2010 | 492 | 2009 | 9 | 59 | 21 | 71 |
| Senegal | 2005 | 376 | 2003 | 9 | 168 | 33 | 192 |
| Senegal | 2010-2011 | 391 | 2006 | 9 | 59 | 57 | 107 |
| Sierra Leone | 2008 | 352 | 2006 | 9 | 59 | 33 | 83 |
| Swaziland | 2006-2007 | 272 | 2002 | 9 | 59 | 57 | 107 |
| Tanzania | 1999 | 176 | NA | NA | NA | NA | NA |
| Tanzania | 2010 | 475 | 2008 | NA | NA | NA | NA |
| Uganda | 2000-2001 | 297 | NA | NA | NA | NA | NA |
| Uganda | 2006 | 368 | 2003 | 6 | 168 | 42 | 204 |
| Uganda | 2011 | 404 | 2009 | 9 | 47 | 33 | 71 |
| Zambia | 2007 | 319 | 2003 | 6 | 180 | 54 | 228 |
| Zimbabwe | 1999 | 228 | 1998 | 9 | 168 | 21 | 180 |
| Zimbabwe | 2005-2006 | 398 | 2002 | 9 | 59 | 45 | 95 |
| Zimbabwe | 2010-2011 | 406 | 2006 | 9 | 59 | 57 | 107 |
| Zimbabwe | 2010-2011 | 406 | 2009 | 9 | 59 | 21 | 71 |
