# Supplementary figures and images for: Transport networks and inequities in vaccination: remoteness shapes measles vaccine coverage and prospects for elimination across Africa
Source: Epidemiol Infect. 2014 Aug 14;143(7):1457–66. doi: 10.1017/S0950268814001988 (PMC4411642; doi:10.1017/S0950268814001988)

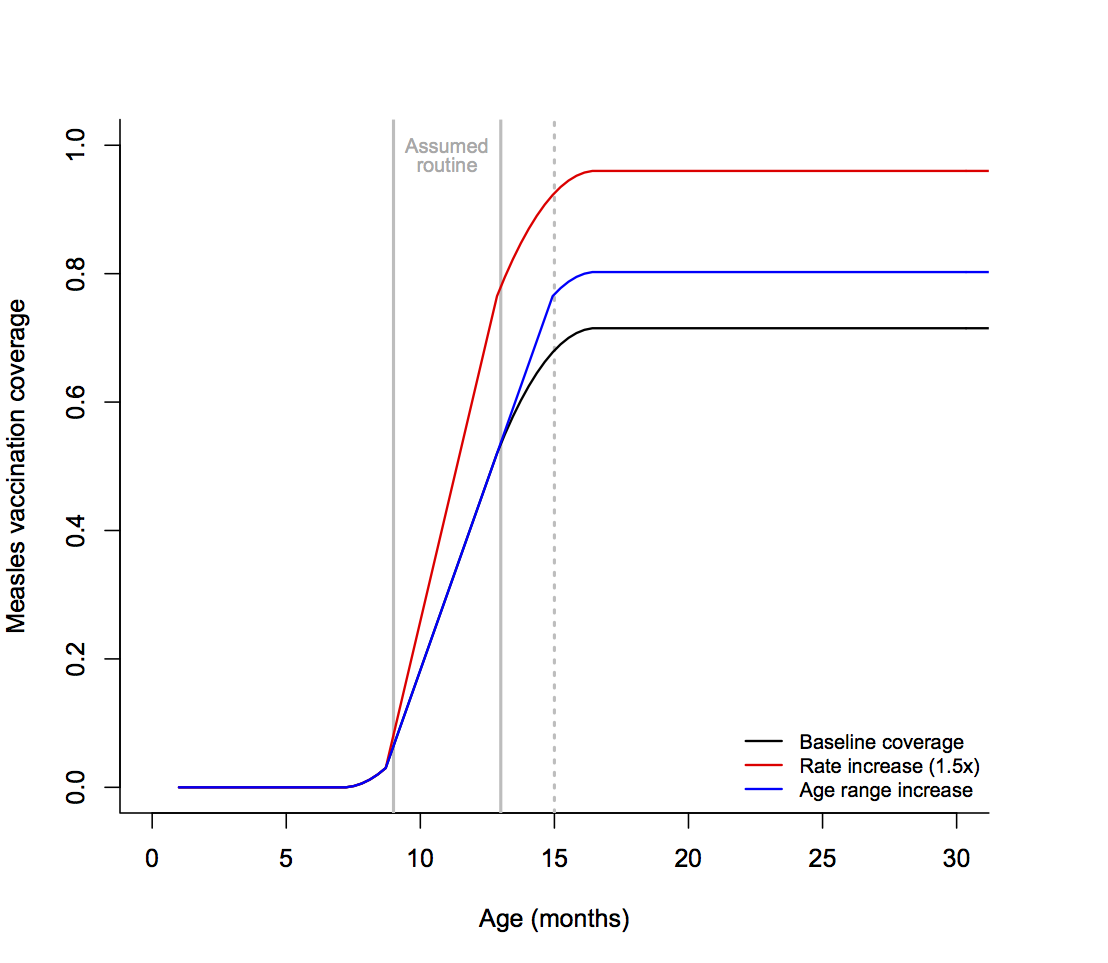

Supplement: Supplementary file 1 [file S0950268814001988sup.zip › S0950268814001988sup001.gif]

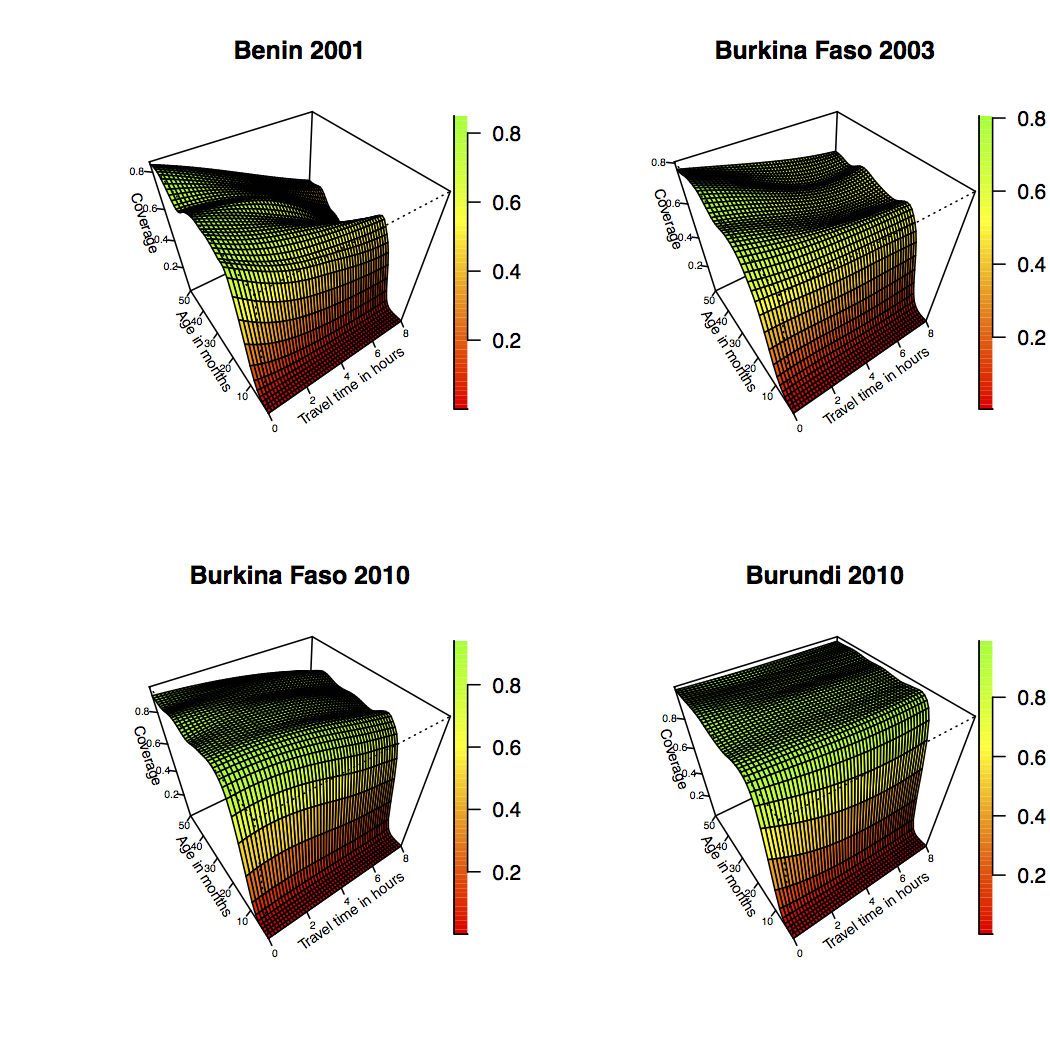

Supplement: Supplementary file 1 [file S0950268814001988sup.zip › S0950268814001988sup002.gif]

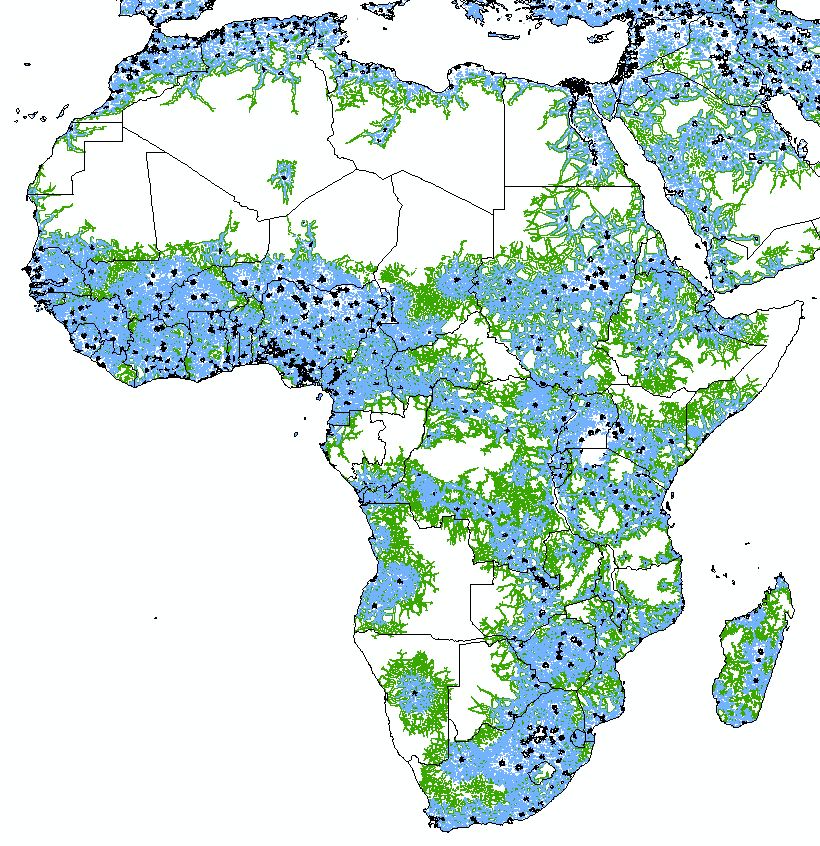

Supplement: Supplementary file 1 [file S0950268814001988sup.zip › S0950268814001988sup003.jpg]

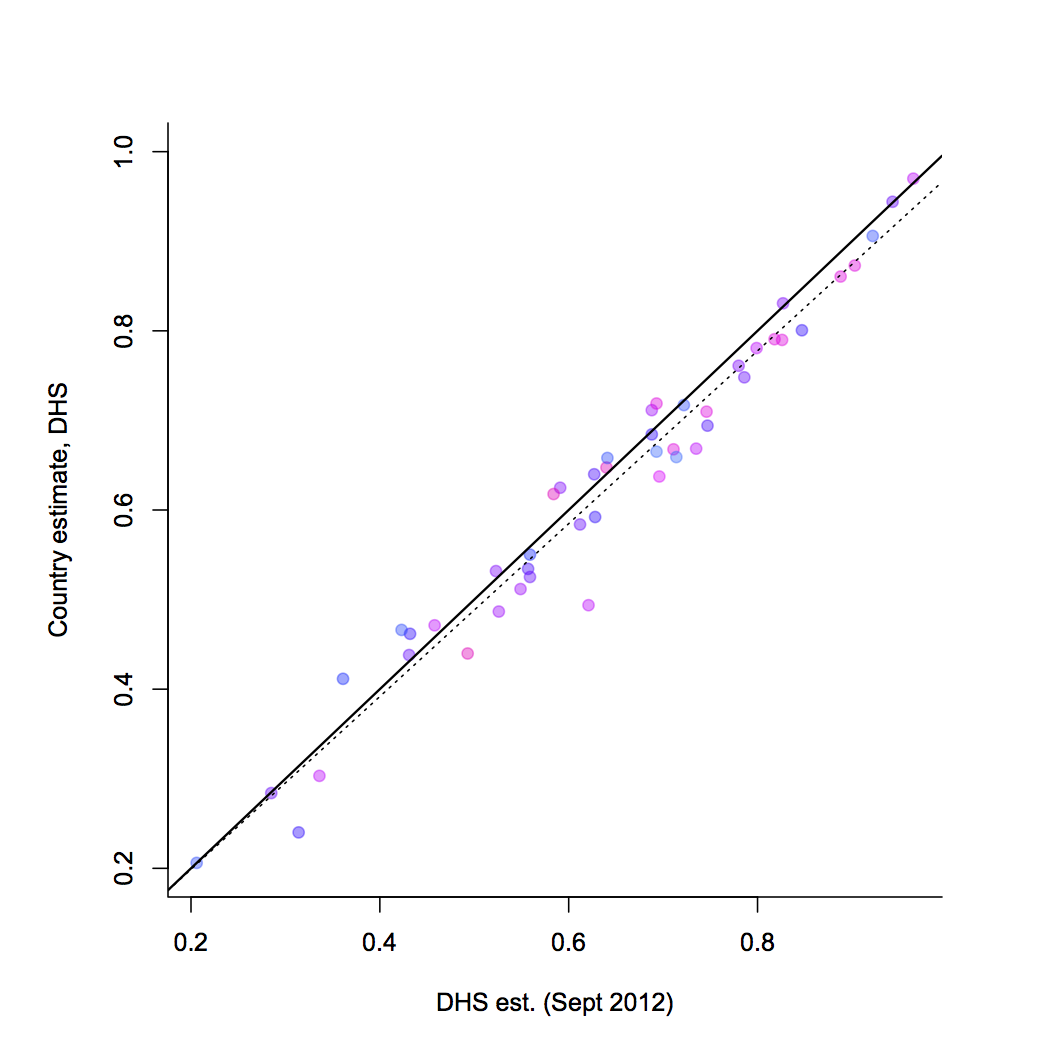

Supplement: Supplementary file 1 [file S0950268814001988sup.zip › S0950268814001988sup004.gif]

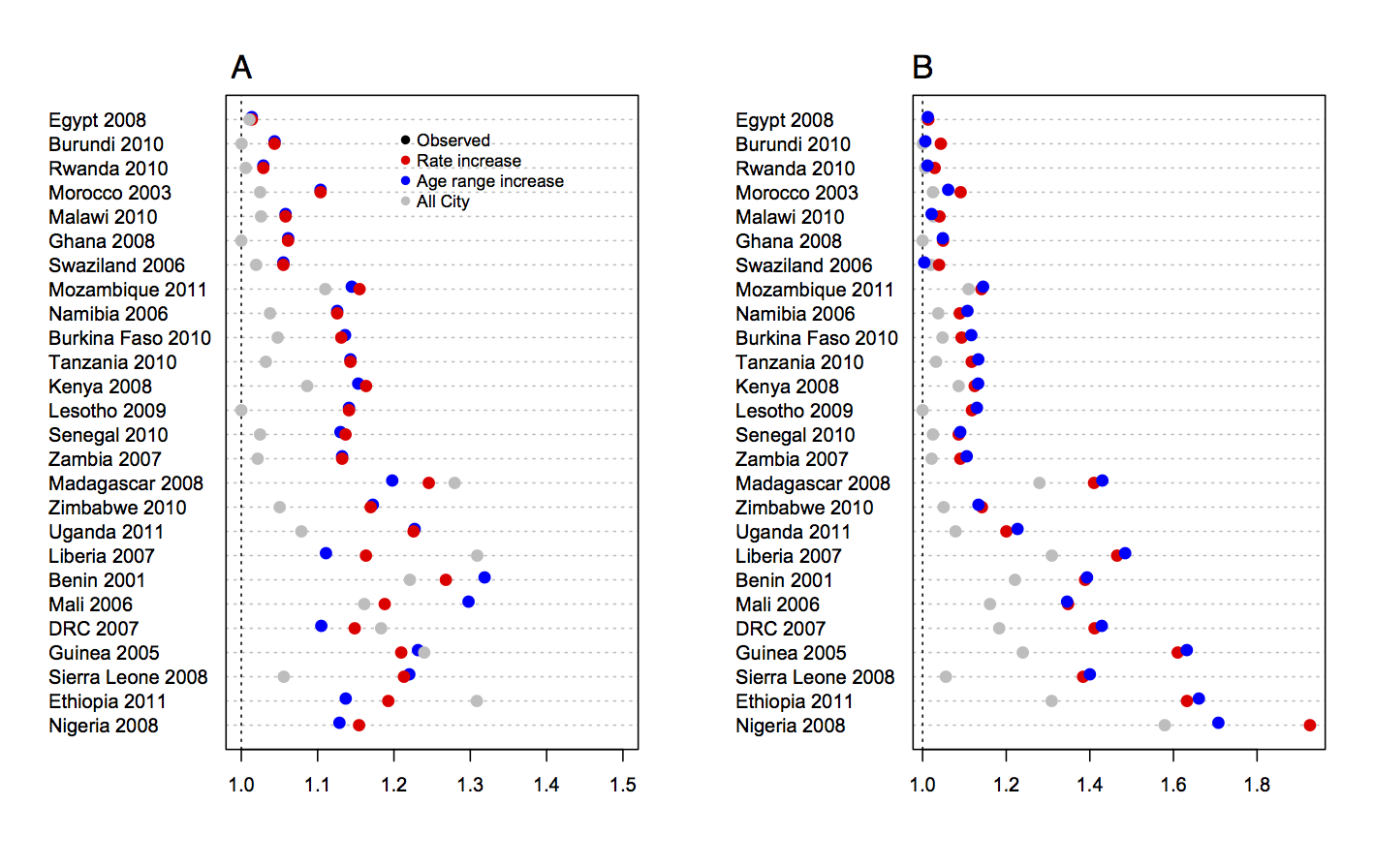

Supplement: Supplementary file 1 [file S0950268814001988sup.zip › S0950268814001988sup005.gif]
